# Photoelectron Imaging Study of the Diplatinum Iodide Dianions [Pt_2_I_6_]^2–^ and [Pt_2_I_8_]^2–^

**DOI:** 10.1021/acs.jpca.2c02008

**Published:** 2022-05-27

**Authors:** Jemma A. Gibbard, Jan R. R. Verlet

**Affiliations:** Department of Chemistry, Durham University, Durham DH1 3LE, United Kingdom

## Abstract

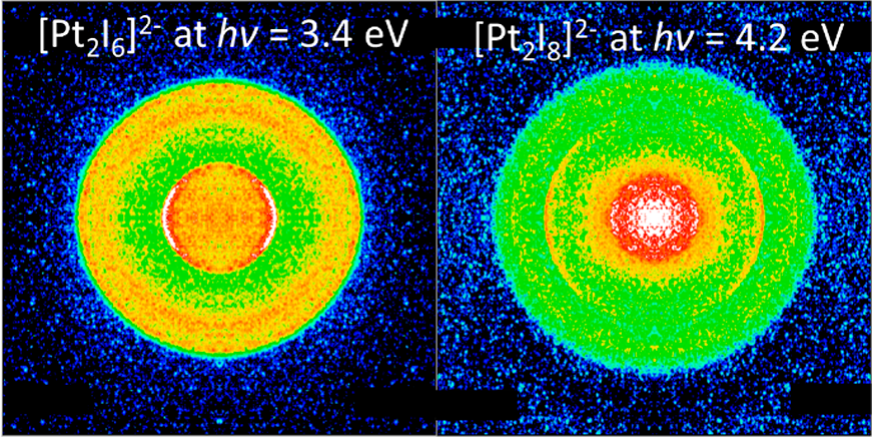

Photoelectron spectroscopy has been used to study the electronic structure, photodetachment, and photodissociation of the stable diplatinum iodide dianions [Pt_2_I_6_]^2–^ and [Pt_2_I_8_]^2–^. Photoelectron spectra over a range of photon energies show the characteristic absence of low kinetic energy photoelectrons expected for dianions as a result of the repulsive Coulomb barrier (RCB). Vertical detachment energies of ∼1.6 and ∼1.9 eV and minimum RCBs of ∼1.2 and ∼1.3 eV are reported for [Pt_2_I_6_]^2–^ and [Pt_2_I_8_]^2–^, respectively. Both of the diplatinum halides exhibit three direct detachment channels with distinct anisotropies, analogous to the previously reported spectra for PtI_2_^–^ and PtI^–^, suggesting a platinum-centered molecular core that dominates the photodetachment. Additionally, evidence for two-photon photodissociation and subsequent photodetachment channels producing I^–^ are observed for both dianions. Finally, an unexplained feature is observed at photon energies around 3 eV, whose origin is considered. Our work highlights the complex electronic structure of the heavy platinum-halide dianions that are characterized by a dense manifold of electronic states.

## Introduction

Platinum halides have complex electronic structures and exhibit unusual bonding, resulting in very high electron affinities (EA), high formal oxidation states on the platinum core, and behavior as superhalogens.^1−4^ These properties contributed to platinum halide dianions being suggested as possible candidates for the smallest stable dianions.^5,6^ While both [PtCl_4_]^2–^ and [PtBr_4_]^2–^ have been experimentally observed, they were found to be electronically metastable with respect to electron loss, with negative second electron affinities of 0.25 and 0.04 eV, respectively.^7^ The increase in electronic stability offered by the increased size of the halogen is interesting, but no platinum iodide dianion complexes have been reported, presumably because these are metastable with respect to iodide loss. Here, we build on these small platinum halides and consider the isolated diplatinum iodide dianions [Pt_2_I_6_]^2–^ and [Pt_2_I_8_]^2–^. For these molecules, there are outstanding questions concerning how the larger and more polarizable iodine and the presence of two excess electrons, as well as the possibility of a platinum–platinum bond, might affect the electronic structure and photochemistry. To answer these questions, we present a photoelectron imaging study of [Pt_2_I_6_]^2–^ and [Pt_2_I_8_]^2–^.

A universal aspect of the electronic structure of polyanions is the presence of a repulsive Coulomb barrier (RCB) that results from the interplay of long-range repulsive forces and short-range attractive forces.^8−13^ Typically the RCB manifests in the photoelectron spectra of polyanions by a characteristic low electron kinetic energy (eKE) cutoff, below which no electrons can be directly emitted. The location of this cutoff corresponds to the minimum height of the RCB. Photoelectron imaging also allows the photoelectron angular distribution (PAD) to be measured. The PAD can encode information about the molecular orbital from which the electron is ejected;^14^ however, this can be challenging to interpret for dianions where the outgoing electron interacts with the resultant anion and instead provides a measure of the RCB anisotropy.^15−17^

Over a number of years, photoelectron spectroscopy has been performed on the platinum halide dianions [PtCl_4_]^2–^, [PtBr_4_]^2–^, [PtCl_6_]^2–^, and [PtBr_6_]^2–^ by Wang and co-workers.^7,10,18,19^ Both [PtCl_4_]^2–^ and [PtBr_4_]^2–^ are metastable while [PtCl_6_]^2–^ and [PtBr_6_]^2–^ are stable with respect to electron loss.^7^ Photoelectron spectroscopy and electronic structure calculations have also been performed on the diplatinum dianion [Pt_2_(μ-P_2_O_5_H_2_)_4_H_2_]^2–^, which indicated a square-planar geometry of ligands around each platinum atom with a Pt–Pt bond, and reported an experimentally measured adiabatic detachment energy (ADE) of 2.2 eV and a minimum height of the RCB of 3.5 eV.^20^ Recently, we used photoelectron spectroscopy at a range of photon energies to study the structure, electron loss, PADs, and photodissociation dynamics of the smallest platinum iodides, PtI_2_^–^ and PtI^–^.^21^ The photoelectron spectra for PtI_2_^–^ and PtI^–^ were very similar to each other and to the previously studied PtCl_2_^–^,^22^ consisting of three direct detachment peaks over a range of ∼1 eV, with distinct anisotropies, indicating photodetachment from a localized Pt–X molecular core. Here, we use a similar methodology to study the photodetachment and photodissociation dynamics of [Pt_2_I_6_]^2–^ and [Pt_2_I_8_]^2–^ as well as report the PADs. Strong correlations are seen between the photoelectron spectra of the diplatinum iodide dianions and the diatomic and triatomic platinum iodide anions, suggesting that the electronic structures of all the platinum iodide species studied to date, whether anionic or dianionic, are similar to the character of the small Pt–I molecular core.

## Experimental Methods

Photoelectron imaging of mass-selected anions was performed at multiple photon energies by using an instrument which has been described in detail elsewhere.^23,24^ Briefly, electrospray ionization of a solution of 2 mM K_2_PtI_6_ in methanol produced the anions, which were desolvated in a capillary. Ring-electrode guides were used to transfer anions through a series of differentially pumped regions and into a ring electrode trap, where they were stored before acceleration and mass selection in a Wiley–McLaren time-of-flight spectrometer. Collision-induced dissociation can also be performed in the ring-electrode guides.^24,25^ Surprisingly, we did not observe PtI_6_^2–^ in the mass spectrum, suggesting that it may be quite unstable as an isolated dianion or metastable with a lifetime shorter than the transition time of the dianion through the instrument (on the many milliseconds range). Given that PtCl_6_^2–^ and PtBr_6_^2–^ are electronically stable, we suggest that PtI_6_^2–^ is unstable with respect to dissociation. The dianions studied here were some of the most abundant peaks in the mass spectrum, along with PtI_2_^–^ and iodine clusters, suggesting that these dianions have a relatively high stability.

Photodetachment occurred at the intersection of a nanosecond laser pulse and a mass-selected dianion packet. Laser pulses with photon energies in the visible and UV were produced via a Nd:YAG pumped optical parametric oscillator. The photoelectrons were imaged on a dual microchannel plate detector in a velocity map imaging configuration,^26^ resulting in an eKE spectrum following deconvolution with a polar onion-peeling algorithm.^27^ Photoelectron imaging also yields the PADs of the emitted electron relative to the laser polarization axis, which was parallel to the detector face. Calibration of the electron spectrometer using the photodetachment of iodide indicated an energy resolution of 5% of the eKE.

Electronic structure calculations were not attempted for [Pt_2_I_6_]^2–^ and [Pt_2_I_8_]^2–^ in this paper. Relativistic effects, the large contribution and complexity of spin–orbit coupling, and an abundance of electronic states motivated us to not perform such calculations on the simpler PtI_2_^–^ and PtI^–^ as we had little confidence in the results. In the present case, electronic structure calculations would be even more challenging as the molecules are larger, may contain metal–metal bonds, and have an extra excess electron.

## Results

Photoelectron spectra were recorded for [Pt_2_I_6_]^2–^ and [Pt_2_I_8_]^2–^ using nanosecond (ns) laser pulses with photon energies, *hν* = 2.8–4.2 eV. All of the photoelectron spectra are reported on an eKE and electron binding energy (eBE) scale, where eBE = *hν* – eKE. Each spectrum is normalized to the most intense feature within that spectrum. From the PADs, an anisotropy parameter (−1 ≤ β_2_ ≤ 2) can be extracted for each spectral feature.^28,29^ Strong similarities are seen between the photoelectron spectra for [Pt_2_I_6_]^2–^ and [Pt_2_I_8_]^2–^, with direct photodetachment and two-photon photodissociation and photodetachment channels observed in both cases. The spectra will be reported here, and the implications for the electronic structure and the photochemistry of the diplatinum iodide dianions will be discussed in the following section.

### [Pt_2_I_6_]^2–^

**Figure 1:**
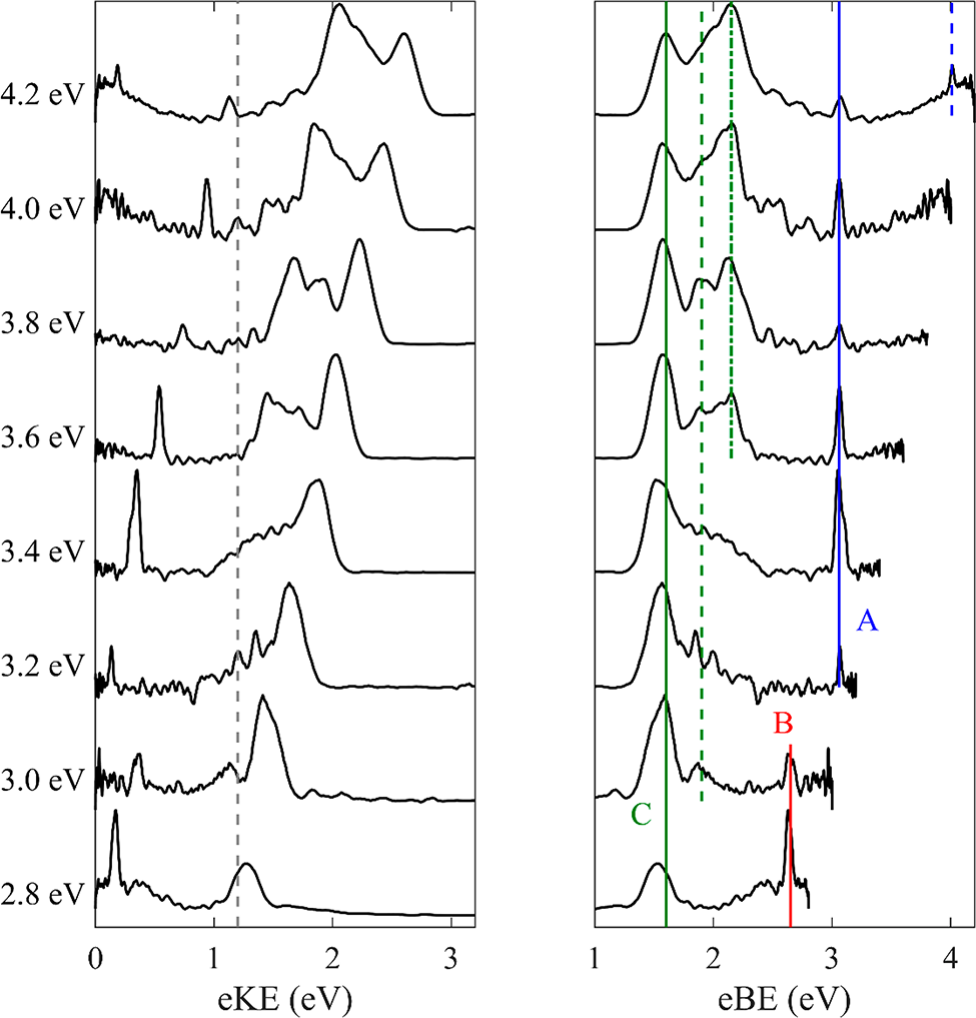
Photoelectron spectra of [Pt_2_I_6_]^2–^ recorded with *hν* = 2.8–4.2 eV on an eKE (left) and an eBE (right) scale. The minimum height of the RCB is shown by a dashed gray line. Direct detachment channels of [Pt_2_I_6_]^2–^ are depicted in green and labeled C with the neutral ground state (solid), first excited (dashed), and second excited (dash-dotted) state shown. Two-photon photodissociation and subsequent photodetachment of I^–^ is shown in blue and labeled A. The peak highlighted in red and labeled B will be discussed in the text.

Figure 1 shows the photoelectron spectra of [Pt_2_I_6_]^2–^ recorded from *hν* = 2.8–4.2 eV, at 0.2 eV intervals. The identity of the ion was determined by its time-of-flight indicating a mass-to-charge ratio of *m*/*z* = 576 amu in combination with the spectral characteristics of a polyanion. On the basis of the natural isotopic abundance of platinum, isotopologues with *m*/*z* between 571 amu ([^190^Pt_2_I_6_]^2–^) and 579 amu ([^198^Pt_2_I_6_]^2–^) are expected, where 576 amu ([^195^Pt_2_I_6_]^2–^) is the most abundant *m*/*z*, but individual isotopologues are not resolved in the mass spectra.

The photoelectron spectra exhibit three distinct features: (A) a sharp peak for *hν* > 3.2 eV with eBE = 3.06 eV (blue), (B) a sharp peak for *hν* < 3.0 eV with eBE = 2.62 eV on a broader background (red), and (C) a broader collection of peaks at higher eKE, which increase in structural complexity and relative intensity as the photon energy increases (green). PtI_3_^–^ is isobaric with [Pt_2_I_6_]^2–^, meaning it may also be present in the mass-selected ion packet. However, the presence of the low-eKE cutoff in photoelectron signal, which is a characteristic of a polyanion, and given that the EA of PtCl_3_^–^ is ∼4.5 eV,^22^ so that there would be insufficient energy to photodetach PtI_3_^–^ at the photon energies used here, indicate that even if it was present, it would not contribute to the spectra shown here.

As previously noted, the onset of the photoelectron signal corresponds to the height of the outer RCB. From Figure 1, we find that this is ∼1.2 eV for [Pt_2_I_6_]^2–^. From the most intense portion of the first direct detachment feature, we find VDE ∼ 1.6 eV for [Pt_2_I_6_]^2–^. The VDE and minimum height of the outer RCB for [Pt_2_I_6_]^2–^ are comparable to previously reported values for [Pt_2_(μ-P_2_O_5_H_2_)_4_H_2_]^2–^ of 2.3 and 1.2 eV (extracted from the reported height of the inner RCB), respectively.^20^

Features A and B appear below the RCB, where no signal should be present. Their appearance could arise from resonant excitation to an excited state from which electrons can be lost by tunneling through the RCB.^15,16,30−36^ Alternatively, they could arise from a two-photon process through the formation of an anionic fragment that is subsequently photodetached. Feature A is a sharp, atomic-like peak with an ADE of 3.06 eV, which allows us to assign it to detachment of I^–^. This is further verified by the ^2^P_1/2_ spin–orbit excited state of I seen at *hv* = 4.2 eV. Iodide is presumably formed via a two-photon process of photodissociation of [Pt_2_I_6_]^2–^ followed by photodetachment of I^–^. As I^–^ is an anion, there is no RCB present, and photoelectrons are not restricted by a cutoff for this photodetachment step. The process by which the I^–^ is formed is, however, not clear. It could occur by Coulomb explosion to form I^–^ and [Pt_2_I_5_]^−^, which would have its own ionic RCB to overcome. This can either take place on an excited state or the ground electronic state of the dianion following some internal conversion process. The dissociation to form I^–^ over such a broad range of photon energies (and likely to also be operable at lower *hv*) would be an indication of a very large density of electronic states of the dianion. Alternatively, I^–^ could be formed by a dissociative photodetachment (DPD) process in which [Pt_2_I_6_]^−^, produced via photodetachment, spontaneously decays to form I^–^ and [Pt_2_I_5_]. As discussed above, it is possible that PtI_3_^–^ is present in the ion beam and able to undergo photodissociation to produce the observed anionic fragments, but this would be impossible to distinguish from photodissociation of [Pt_2_I_6_]^2–^ using our experimental apparatus.

At and below *hν* = 3.0 eV there is insufficient photon energy to photodetach I^–^, but a sharp feature at low eKE on top of a broad background signal is also observed for these photon energies (but not at higher *hν*). This feature again has the appearance of an atomic photoelectron spectrum with an eBE of 2.62 eV. The origin of this feature is unclear as its eBE does not correlate with the electron affinity of any known atomic element, let alone I or Pt. Potential explanations for the feature will be explored in the Discussion section.

At the highest photon energies studied, up to three distinct peaks with different PADs are observable above the RCB. These features shift to higher eKE as the photon energy is increased, suggesting a direct detachment mechanism. The direct detachment features are very reminiscent of the structure of the direct detachment channels of PtI^–^ and PtI_2_^–^.^21^ At *hν* = 4.0 eV the PAD is characterized by β_2_ = −0.9, −0.6, and 1.5 for electronic states with increasing eBE. Interpreting the PADs following direct detachment of a dianion cannot be done in the same manner as for a monoanion, as the location of the remaining excess negative charge ultimately determines the final PAD and therefore must be accounted for.^8,15,16^ In the absence of high-level electronic structure calculations for [Pt_2_I_6_]^2–^, it is difficult to extract any information about the nature of the molecular orbitals from the PAD, other than to say the presence of a number of electronic states with differing anisotropy appears to be a characteristic of platinum iodides, as both singly and doubly charged anions.

### [Pt_2_I_8_]^2–^

**Figure 2:**
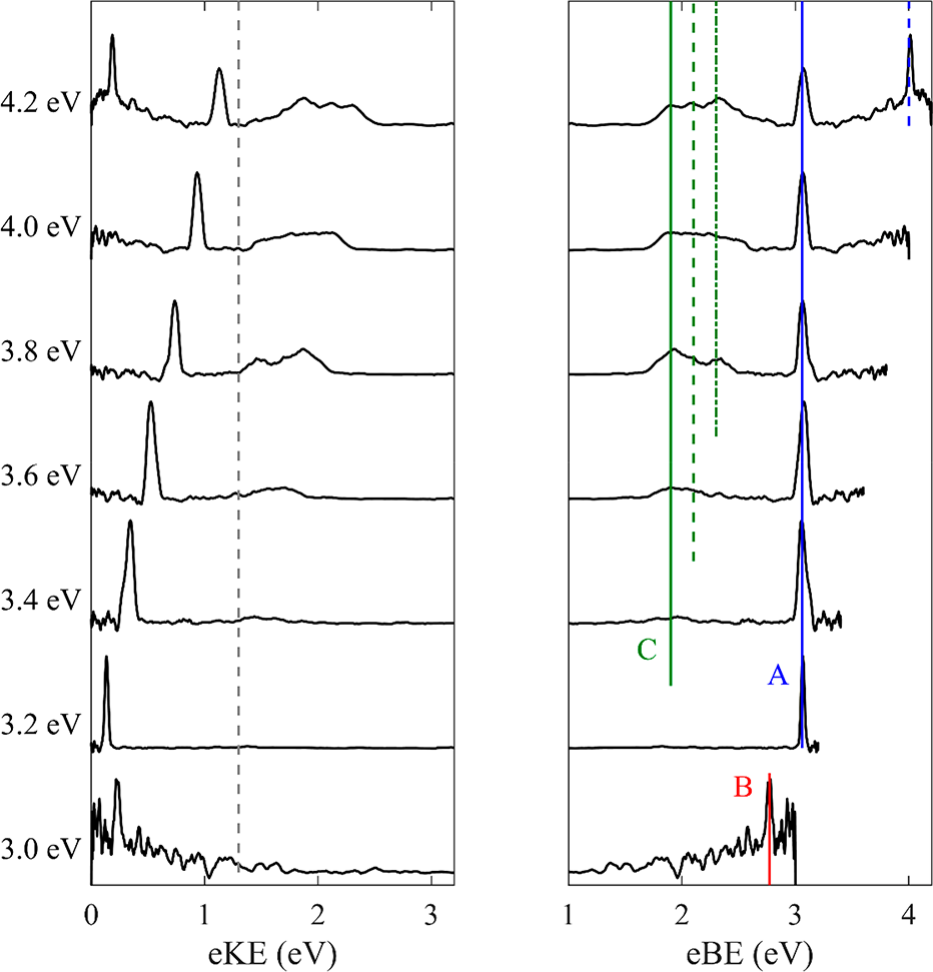
Photoelectron spectra of [Pt_2_I_8_]^2–^ recorded with *hν* = 3.0–4.2 eV on an eKE and eBE scale. Direct detachment channels of [Pt_2_I_8_]^2–^ are depicted in green and labeled C with the neutral ground state (solid), first excited (dashed), and second excited (dash-dotted) state shown. The minimum height of the RCB is shown by a dashed gray line. Two-photon photodissociation as well as subsequent photodetachment of I^–^ is shown in blue (solid for ^2^P_3/2_ and dashed for ^2^P_1/2_) and labeled A. The peak highlighted in red and labeled B will be discussed in the text.

Figure 2 shows the photoelectron spectra of the ion packet with *m*/*z* ∼ 703 amu recorded with a nanosecond laser between *hν* = 3.0–4.2 eV. Given the *m*/*z* and that the spectra share many similarities with those for [Pt_2_I_6_]^2–^, this ion most probably corresponds to [Pt_2_I_8_]^2–^, which has an expected isotopologue distribution between *m*/*z* = 698 and 706 amu. Again, three clear features are present: (A) a low-eKE feature attributable to the photodetachment of I^–^ (blue); (B) a sharp feature on a broad background at eBE = 2.77 eV (red); and (C) a higher energy broad feature consisting of multiple peaks from direct detachment of the parent anion (green). In this case the relative intensity of the two-photon feature is much larger than the one-photon direct detachment channel, at all photon energies. The minimum height of the outer RCB is estimated to be ∼1.3 eV. The VDE is measured to be ∼1.9 eV and comparable with the VDEs of [Pt_2_I_6_]^2–^ and the previously reported [Pt_2_(μ-P_2_O_5_H_2_)_4_H_2_]^2−^. Similar to the case of [Pt_2_I_6_]^2–^, PtI_4_^–^ is isobaric with [Pt_2_I_8_]^2–^ and could be present in the ion beam. However, the spectra indicate a dianion and the EA for PtCl_4_^–^ and PtF_4_^–^ are higher than the photon energies used here (EA ≥ 5 eV),^22,37^ suggesting that even if present, PtI_4_^–^ would not photodetach.

The low-eKE feature (blue line in Figure 2) is again attributable to a two-photon process of photodissociation and subsequent photodetachment of the resulting I^–^ fragment, similar to the observation for [Pt_2_I_6_]^2–^ (blue line in Figure 1). Unlike [Pt_2_I_6_]^2–^, this is the dominant channel at all photon energies above *hν* ≥ 3.2 eV (i.e., where it can be probed by the photon energy used). The relative intensity of feature A to that of C decreases with increasing *hν*. This may indicate a decrease in the photodissociation or photodetachment cross section in the ionic fragmentation channels, but it is more likely attributed to the direct detachment of the dianion becoming energetically accessible at the photon energy used. At the highest photon energy used, there is sufficient energy to photodetach to both spin–orbit states of I, resulting in the pair of sharp peaks separated by 0.95 eV. As before, I^–^ is observed over a very large excitation range, and we do not see evidence of electron tunneling through the RCB, although there are clearly excited states accessed.^38^ As PtI_4_^–^ may be present in the ion beam, it could undergo photodissociation and be the source of the observed I^–^.

Similar to [Pt_2_I_6_]^2–^, below *hν* = 3.0 eV a second sharp feature on a broad background labeled B is observed with eBE = 2.77 eV (red). In this case, it is unclear whether the feature shifts linearly with *hν*, as it is only present at one photon energy studied and we note that it is exceedingly weak. Nevertheless, it has the appearance of an atomic anion, but, as before, no element has the matching electron affinity.

The high-eKE feature, which is strongly affected by the presence of the RCB, originates from direct photodetachment of [Pt_2_I_8_]^2–^. Similar to the previous platinum iodide species, three electronic states are observed in the spectra at higher *hν*, but they are difficult to conclusively discern as the spectral width of the feature increases with photon energy, too. Distinct PADs are observed for the three features present at *hν* = 4.2 eV characterized by β_2_ = −1.0, −0.5, and 1.0 for states with increasing eBE. Similar to [Pt_2_I_6_]^2–^, it is challenging to interpret the PADs for [Pt_2_I_8_]^2–^, although we note that the overall trend of β_2_ < 0, < 0, and > 0 was also observed for [Pt_2_I_6_]^2–^, thus suggesting a similar overall electronic structure.

## Discussion

In this section the results for [Pt_2_I_6_]^2–^ and [Pt_2_I_8_]^2–^ will be compared and contrasted with each other and with previously studied platinum halides and other platinum clusters. The photoelectron spectra for [Pt_2_I_6_]^2–^ and [Pt_2_I_8_]^2–^ exhibit a number of similarities including multiple direct detachment features with strongly anisotropic PADs (feature C, green lines in Figures 1 and 2) and photodissociation channels producing I^–^ (feature A, blue in Figures 1 and 2). Additionally, the photoelectron spectra of all of the platinum iodides studied here contain extra features indicative of other anionic fragments formed via photodissociation of the parent species (feature B, red in Figures 1 and 2). Strong similarities are also observed between the direct detachment channels observed here (feature C, green lines in Figures 1 and 2) and those for other platinum iodide anions and platinum halide anions more generally.

Assuming the negative charges are localized on the iodine ligands, then the oxidation state of platinum is +2 (d^8^) in [Pt_2_I_6_]^2–^ and +3 (d^7^) in [Pt_2_I_8_]^2–^. The constituent PtI_3_^–^ and PtI_4_^–^ fragments are electron-deficient with 14 and 15 electrons, respectively, in contrast to the 18-electron count of many stable transition metal complexes. Dimers are likely to form to stabilize the electron-deficient fragments, either by the formation of a platinum–platinum bond or via bridging iodine ligands. Given that in the solid phase [Pt_2_I_6_]^2–^ and [Pt_2_I_8_]^2–^ demonstrate a preference for platinum cores linked by bridging iodine atoms, as determined via X-ray diffraction of solid state crystals with various counterions, a similar structure may be expected in the gas phase, too.^39,40^ Additionally, the crystal structures of the dianions indicate square-planar and octahedral coordination geometries of iodine ligands around the platinum atoms, which are also likely in the gas phase.^39,40^ While photoelectron spectroscopy is a powerful tool for studying the electronic structure of molecules, it is less well-suited to obtain structural information, especially in the absence of calculations. Infrared spectroscopy would be more suitable for studying the nuclear geometry of these diplatinum dianions in the gas phase.

The observed VDEs for [Pt_2_I_6_]^2–^ and [Pt_2_I_8_]^2–^ are similar in magnitude to each other (1.6 and 1.9 eV) but significantly smaller than for PtI_2_^–^ (3.5 eV). This difference between the dianions and anions is due to the larger electron–electron repulsion in the dianions. The marginally higher RCB observed for [Pt_2_I_8_]^2–^ compared to [Pt_2_I_6_]^2–^ (∼1.3 and ∼1.2 eV) is somewhat unexpected given the larger size of [Pt_2_I_8_]^2–^, which may be expected to facilitate greater separation of the excess charges and reduce the Coulomb repulsion within the molecule. However, this type of argument is strongly dependent on the geometries of the dianions, which are not determined here. The VDEs and minimum heights of the outer RCBs we record for [Pt_2_I_6_]^2–^ and [Pt_2_I_8_]^2–^ are similar to values previously recorded for [Pt_2_(μ-P_2_O_5_H_2_)_4_H_2_]^2–^ of 2.3 and 1.2 eV (inner RCB is 3.5 eV), respectively.

The motif of three sharp peaks in the direct detachment feature with an overall spectral width of ∼1 eV and with different anisotropies is present in both of the dianions studied here as well as the previously investigated PtI_2_^–^ and PtI^–^.^21^ For the latter, the three-band motif and the observed PAD for those anions (β_2_ < 0, > 0, and > 0 with increasing eBE) were explained by using a simple d-block model of the Pt–I bond. The strong similarity of the structure of feature C in the dianion spectra suggests that this motif is a general feature of the platinum–iodine (halogen) bond. The similarity of [Pt_2_I_6_]^2–^ and [Pt_2_I_8_]^2–^ to the smaller anions suggests that these are highly symmetrical molecules, where direct detachment is occurring from a small molecular core. This result may indicate that the dimers are linked via bridging iodides similar to the known solid state crystal structures,^39,40^ rather than a platinum–platinum bond, due to the strong spectral similarities between the diplatinum iodide dianions and the platinum iodide anions. However, it should be noted that this similarity is also evidence of the limited scope photoelectron spectroscopy has to unravel the full geometric structure of the diplatinum dianions, as the three observed transitions can arise from just a single Pt–I bond.

As noted previously, we have not attempted electronic structure calculations for [Pt_2_I_6_]^2–^ and [Pt_2_I_8_]^2–^. It is very challenging to perform such calculations of small platinum halides, such as PtI_2_^–^ and PtI^–^, due to the large spin–orbit coupling of both Pt and I as well as the substantial relativistic effects that need to be accounted for.^21^ On top of this, the diplatinum iodide dianions have the challenges of containing more atoms and the need to accurately account for the presence of two excess electrons within the dianion’s molecular framework. A simple d-block model can be used to attempt to understand the nature of the highest energy MOs by considering symmetry.^41^ However, spin multiplicities and spin–orbit coupling, particularly *J*–*J* coupling which is used for Pt, complicate the picture greatly by adding many additional states, making even a qualitative interpretation challenging. Hence, we prefer to not endeavor into such speculation here.

The PADs of the dianions are dictated by two dominant factors: the location of negative partial charges in the remaining anion and the transition dipole vector associated with detachment.^15,16,42,43^ The fact that the three different features have different PADs is likely associated with differences in the transition moments. The PADs could offer new insights into the geometric structure of the dianion but would require accurate electronic structure calculations to relate the RCBs to the PADs.^8,11,15,36^

The diplatinum iodides undergo photodissociation to form I^–^, as evidenced by the presence of electrons from the photodetachment of I^–^ at specific photon energies. The mechanism for I^–^ production is unclear and could derive from a Coulomb explosion in the excited state, DPD via an excited dianion state, or rapid internal conversion from the excited state to the electronic ground state, where the presence of substantial internal excitation leads to iodide loss. As there are likely to be many excited dianion states and the relative intensity of the I^–^ feature compared to direct detachment (features A vs C in Figures 1 and 2) changes with *hν*, it seems more likely that dissociation occurs via an excited state. The production of photoelectrons from I^–^ is a two-photon process of photodissociation and subsequent photodetachment. For all of the species this channel is visible at photon energies below the photodetachment threshold of the platinum iodide complex. The relative intensity of feature A decreases once photodetachment becomes an open channel. The relative intensity of features C, compared to feature A, is much larger for [Pt_2_I_8_]^2–^ than [Pt_2_I_6_]^2–^, indicating that the larger cluster has a stronger preference for photodissociation than electron loss. This could be explained by the increased strain in the larger cluster acting as a driving force for I^–^ loss rather than photodetachment.

One outstanding question concerns the nature of the sharp atom-like feature below *hν* < 3.0 eV for the diplatinum dianions (red line in Figures 1 and 2). For both species, there is a sharp peak on top of a broader background with eBE = 2.62 and 2.77 eV for [Pt_2_I_6_]^2–^ and [Pt_2_I_8_]^2–^, respectively. The photoelectron emission of this feature is also highly anisotropic with β_2_ = 1.0 for [Pt_2_I_6_]^2–^. Because of the lower signal levels, it was not possible to confidently extract a β_2_ for [Pt_2_I_8_]^2–^. It is observed for multiple photon energies for [Pt_2_I_6_]^2–^ and shifts by the change in photon energy, suggesting direct detachment. The sharp nature might suggest atomic emission; however, the binding energies do not correlate with any atom (only the halogens have electron affinities that exceed 2.5 eV). Finally, we note that it appears to be a two-photon feature because it arises below the cutoff imposed by the RCB of the dianion. The signal was too weak to perform power-dependent studies to absolutely verify this.

Given the above observation, a more detailed discussion appears appropriate. One possible explanation is that photoelectrons are tunneling through the RCB, which is typically characterized by a feature that can be anisotropic (if tunneling detachment is faster than rotational dynamics).^15,16,34^ However, this seems unlikely because, while such peaks have been observed, they have never been as narrow as seen here and not at such low eKEs. The low eKE would suggest that an electron would be tunneling through a very wide and high barrier (∼1 eV), which would not be fast and therefore would not lead to an anisotropic nor necessarily a narrow distribution. Moreover, previous observations of tunneling dynamics were characterized by a feature with an eKE that does not depend on *hv*, which again is not consistent with observations in Figure 1.^35,32^

An alternative explanation is that photodetachment or photodissociation produces a cluster which behaves as a solvated I^–^ and this is subsequently photodetached. Solvation of I^–^ with atoms or molecules typically leads to an increase in VDE as the bonding in the anion is stronger than that in the neutral. But this need not be the case for more strongly bonded systems, and one could quite easily imagine a case in which the final state is more bound than the initial state. Such a process would necessarily be in the anion as an RCB would invalidate this process. Hence, the process could, for example, be
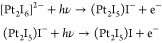
1where the (Pt_2_I_5_)I^–^ complex has absorbed an additional photon to result in the observed feature. We have, however, no evidence to support this suggestion.

A third possible source is that, following photodissociation, the I^–^ is still within the “sphere of influence” of the other anionic fragments and therefore experiencing repulsion from the RCB against dissociation, which lowers the eBE of I^–^. This could explain the additional broad background observed at low eKE, but it is challenging to see how it would result in a sharp feature given the nature of the dynamics of such a process. Finally, we cannot discount that this feature arises from the photochemistry of PtI_3_^–^ and PtI_4_^–^, even though no direct evidence for the presence of these ions is found in this study, and it would be difficult to reconcile why they would have such similar binding energies. At this stage, we cannot conclusively assign the origin of this feature.

## Conclusions

The electronic structure, photodetachment, and photodissociation dynamics of [Pt_2_I_6_]^2–^ and [Pt_2_I_8_]^2–^ have been studied by using frequency-resolved photoelectron imaging. Direct detachment channels are observed as a band of three peaks, with distinct anisotropies and a spectral width of ∼1 eV, which are strongly reminiscent of the photoelectron spectra of PtI_2_^–^ and PtI^–^, suggesting that the highest energy MOs, which contribute to the photodetachment dynamics, are associated with Pt–I bonds. Photodissociation of the diplatinum dianion to produce I^–^, which is photodetached in a two-photon process, is observed for both species studied and dominates at the lower photon energies investigated. An additional two-photon atom-like feature is observed at the lowest photon energies studied, but the origin is unclear. Overall, we show that the photochemistry of the diplatinum iodide dianions is very rich with differing photodetachment and photodissociation channels. While the electronic structure is very complex and would require electronic structure calculations that account for relativistic effects, aspects are also rather simple and can be correlated to that of the Pt–I^–^ photodetachment. We hope our work will stimulate computational interest in these rich systems to further understand their electronic structure and uncover their geometric structure.
